# PW02-020 - Colitis revealing mevalonate kinase deficiency

**DOI:** 10.1186/1546-0096-11-S1-A160

**Published:** 2013-11-08

**Authors:** L Michael, J Camille, BM Brigitte

**Affiliations:** 1 Hopital Robert Debré, Paris, France; 2 CHI Créteil, Créteil, France; 3 Rhumatology, Hôpital Necker Enfants Malades, Paris, France

## Introduction

Hyperimmunoglobulinemia D (HIDS) is the less severe form of mevalonate kinase deficiency (MKD) caused by recessive inherited mutation in the mevalonate kinase gene (*MVK*). HIDS is characterized by febrile attacks, often associated with transient digestive manifestations, such as abdominal pain, diarrhea and vomiting.

## Case report

Here we report for the first time two patients with MKD revealed by a severe neonatal colitis. Both patients had chronic bloody diarrhea and failure to thrive, one patient since the age of one month and the other twelve days. Total parenteral nutrition was required. A marked elevation of acute phase reactants was present, and no evidence of infection was found. In patient 1, ileocolonoscopy revealed an ulcerative colitis at the age of 5 months. Patient 2 suffered from enterocolitis and shock, associated with multiple bowel adhesions at age 5 weeks; the rectosigmoidoscopy showed aphtoid lesions of the sigmoid colon. Pathological findings of colonic biopsies revealed a dense polymorph inflammatory infiltrate associated with deep ulcerations. Febrile attacks occurred 2 months after the onset of digestive symptoms in patient 1, and at onset of disease in patient 2. Genomic sequencing of the *MVK* gene revealed compound heterozygous mutations in both patients. Anti-interleukin 1 (Anti-IL1) agent (anakinra) produced long-term remission of all digestive features and laboratory parameters.

## Discussion

This report emphasizes that MKD may be the cause of severe early-onset inflammatory colitis, and must be considered by physicians, even in the absence of fever, after ruling out infections. Anti-IL1 therapy may result in a dramatic improvement of MKD-related inflammatory bowel disease.

## Disclosure of interest

None declared.
